# Analysis of Upper-Limb Movement Characteristics in Tennis Volleys Based on Skill-Level Differences: Kinematic Features of the Backhand Versus Forehand Volley

**DOI:** 10.3390/jfmk11020203

**Published:** 2026-05-22

**Authors:** Kohei Katsumi, Hitoshi Koda, Noriyuki Kida

**Affiliations:** 1 Master’s Programs of Applied Biology, Graduate School of Science and Technology, Kyoto Institute of Technology, Hashikami-cho, Matsugasaki, Sakyo-ku, Kyoto 606-8585, Japan; kouteni513@gmail.com; 2 Faculty of Arts and Sciences, Kyoto Institute of Technology, Hashikami-cho, Matsugasaki, Sakyo-ku, Kyoto 606-8585, Japan; kida@kit.ac.jp

**Keywords:** tennis, forehand volley, backhand volley, three-dimensional motion analysis, twist, performance analysis

## Abstract

**Background**: In tennis, the volley is an important shot; however, studies describing its movement characteristics have been limited to the forehand volley (FV). In this study, we analyzed the characteristics of upper-limb movement during FV and backhand volley (BV) in skilled and less-skilled tennis players. **Methods**: Twelve tennis players with experience (skilled group) and eight with little experience (less-skilled group) were included. The participants stood in front of a simulated net and volleyed balls were fed toward the target. Movements were recorded using three video cameras, and three-dimensional coordinates were obtained using the direct linear transformation method. The measured variables were bilateral shoulder rotation angle, pelvic rotation angle, shoulder–pelvis twist angle, and the racket–forearm angle. A two-way analysis of variance (ANOVA) was conducted with player level (skilled vs. less skilled) and time point (backswing event vs. impact event) as factors. **Results**: In the FV, a significant main effect of time point was observed for the bilateral shoulder rotation angle (F1,18 = 7.471, *p* = 0.014, η^2^ = 0.293). In the BV, significant main effects at both player level and time point were observed for the pelvic rotation (player level; F_1,18_ = 8.759, *p* = 0.008, η^2^ = 0.327, time point; F_1,18_ = 13.351, *p* = 0.002, η^2^ = 0.426). Also, significant main effects at both player level and time point were observed for racket–forearm angles (player level; F_1,18_ = 6.752, *p* = 0.018, η^2^ = 0.273, time point; F_1,18_ = 10.213, *p* = 0.005, η^2^ = 0.362). However, a significant main effect of the player level was observed for the shoulder–pelvis twist angle (F_1,18_ = 12.124, *p* = 0.003, η^2^ = 0.402). **Conclusions**: In contrast to FV, BV prioritizes ball control by maintaining the shoulder–pelvis angular relationship without releasing the twist. These results suggest that skill-related differences in volleying are more pronounced in the BV than in the FV.

## 1. Introduction

In tennis, a volley is a stroke performed near the net without allowing the ball to bounce. Therefore, it is important to put opponents under time and psychological pressure and control the flow of the match [1,2]. Furthermore, compared to groundstrokes, which are hit from a position away from the net after the ball bounces, the volley is a technique associated with more aggressive tactics aimed at winning points [3]. Generally, volleys are categorized into forehand volleys (FV), performed on the dominant side of the body, and backhand volleys (BV), performed on the non-dominant side of the body. In the FV, the ball is pushed in front of the body on the dominant side, which tends to allow a relatively large upper limb range of motion and trunk rotation. It demands decisiveness in scoring and the ability to generate power, making it a technique for winning points. In the BV, the ball is in contact with the non-dominant side of the body, which tends to limit the upper limb range of motion and trunk rotation. It is important to keep the rally going and reliably return the ball to the opponent’s court [4], making it a technique for avoiding unforced errors and not giving away points.

However, volleying is a skill with which many players tend to feel uncomfortable and is reported to be particularly difficult to master at lower levels of tennis competitions [5]. Although volley technique coaching manuals share common elements, they do not provide a unified set of instructional guidelines. Mitsuhashi and Yamada (2010) reported that an analysis of major instructional manuals revealed that shoulder or pelvic rotation, keeping the racket upright, and compact racket movements are commonly emphasized for volleys [6]. However, interpretations regarding body rotation lack consistency, with manuals stating that the player should turn the body sideways [7], rotate the shoulders [8], or not twist the body [9]. Similarly, with respect to keeping the racket upright, different manuals describe it as L-shaped [10] or at 90° [11]. In addition, regarding the racket action, manuals state that the player should use little or no backswing [12] and set the racket near the shoulder [13]. This suggests that, although coaching manuals share common views on some elements of the volley technique, they differ in how each movement is understood. These findings highlight the need for technical instructions based on scientific evidence.

Previous studies have focused on improving tennis performance. In particular, research using three-dimensional motion analysis has focused on the aspects of movement patterns and stroke forms that have not yet been clarified. By quantitatively describing these aspects based on three-dimensional coordinates, this research provides kinematic and kinetic insights. These findings have been used to enhance player performance and prevent injuries [14,15]. Studies have reported analyses of lower limb movements and shoulder joint kinematics in top-level players [16], as well as investigations into differentiating serve types [17]. Studies have examined the fundamental aspects of footwork [18] and the relationship between stroke velocity adjustments and ground reaction forces [19]. These studies indicate that quantifying movement is key to improving techniques, and three-dimensional motion analysis is likely to be similarly effective for volleying.

However, research on motion analysis that specifically targets volleys is limited. The only motion analysis study on volleys has reported an upper-limb kinematic analysis focusing on the FV [20]. This study reports how skill level affects the bilateral shoulder rotation angle during the volley, racket–forearm angle, and relative angle of the racket’s center point with respect to the upper trunk. The research is limited to FV and is insufficient for understanding the overall volley mechanics. Despite the technical difficulties of BV, there has been little kinematic research on this type of stroke. In fact, because BV is performed on the non-dominant side of the body, the structure of the upper limb kinetic chain and trunk use may be fundamentally different from those of FV. Furthermore, limitations in the range of motion of the shoulder and elbow joints, along with reduced coordination on the nondominant side, may affect movement stability [21]. However, these possibilities remain speculative and scientific analysis is needed to objectively clarify the differences between FV and BV.

In sports biomechanics, comparison of skilled and less-skilled individuals is important for understanding movement mechanics. Under time-constrained conditions, the differences in skill levels become more evident [22,23]. In this context, the tennis volley is a highly time-constrained stroke in which differences in movement coordination and stability associated with skill level are likely to become particularly pronounced. Previous studies have shown that the rotation angles of the pelvic girdles are important factors influencing ball speed and spin in tennis strokes [24,25]. However, these kinematic characteristics have not been examined in tennis volleys.

It is important to examine differences in the movement characteristics of FV and BV between skilled and less-skilled players. For BV, where kinematic evidence remains limited, clarifying upper limb coordination patterns and how these characteristics differ by skill level may contribute to the development of more practical coaching guidelines. Accordingly, the purpose of this study was to investigate FV and BV in skilled and less-skilled players and to examine the kinematic characteristics of BV.

## 2. Materials and Methods

### 2.1. Participants

The participants comprised 12 male university students (21.0 ± 0.91 years) who were members of the varsity tennis team and eight male university students (22.1 ± 1.96 years) who had 6 months or less of tennis experience. The former was classified as the skilled group (ten right-handed, two left-handed), and the latter was classified as the less-skilled group (seven right-handed, one left-handed). The skilled group in the present study consisted of university varsity players competing mainly at the regional level; therefore, the term “skilled” should be interpreted relative to the novice group and not as indicating national-level performance. The study was conducted in accordance with the Declaration of Helsinki, and the protocol was approved by the Kyoto Institute of Technology Ethics Committee for Scientific Research Involving Human Subjects (Approval No. 2024-39). All participants received verbal explanations regarding the study objectives, data usage, and protection of personal information, and written informed consent was obtained prior to participation.

### 2.2. Experimental Task

**Figure 1:**
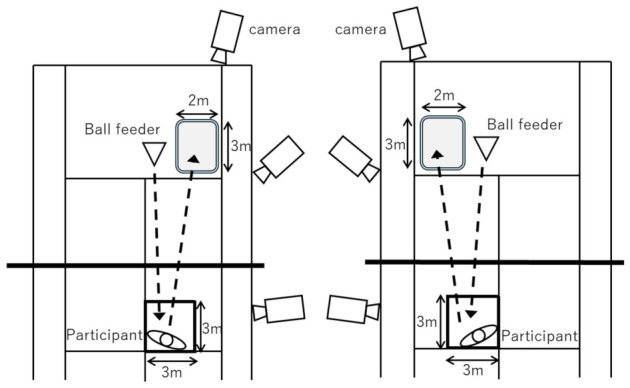
Schematic diagram of the experimental setup for the upper-limb trials. The (**left**) and (**right**) figures show the forehand volley and the backhand volley, respectively. The participants performed multiple middle volleys toward the 3 m × 2 m target area on the opposite side of the net using balls fed at 50–60 km/h; one trial that the participant judged satisfactory was selected for analysis and recorded with a video camera. Three cameras were positioned so that the reflective markers could be captured from different directions for three-dimensional reconstruction, while avoiding interference with task performance.

The experiments were conducted in a gymnasium. After the warm-up, the participants were positioned 4 m behind a simulated net (1 m high), as shown in Figure 1.

They were then instructed to volley balls fed by a ball feeder from a position 8 m behind the net on the opposite side of the court. The balls were fed to facilitate middle volleys, and the participants were instructed to direct the volleys toward a 3 m × 2 m target area. The initial ball-feeding speed was controlled within the range of 50–60 km/h and was measured using a speed gun (Yupiteru 16JYM11000, Mizuno, Osaka, Japan). For each participant and condition, one valid trial was selected for analysis to represent a successfully executed volley. Trials with obvious mishits or failure to complete the task as intended were excluded. Although averaging multiple valid trials would provide a more reliable estimate of performance, the present study adopted a single-trial approach as an exploratory analysis of volley kinematics. Under these conditions, the FV and BV trials of the participants were recorded using video cameras (JVC GC-YJ40; Victor Company of Japan, Limited; Yokohama, Japan) at 59.94 fps with an f-stop of 2.8. Before data collection, the recording conditions were adjusted to ensure that the attached markers were clearly visible as points in the video images.

### 2.3. Data Acquisition

**Figure 2:**
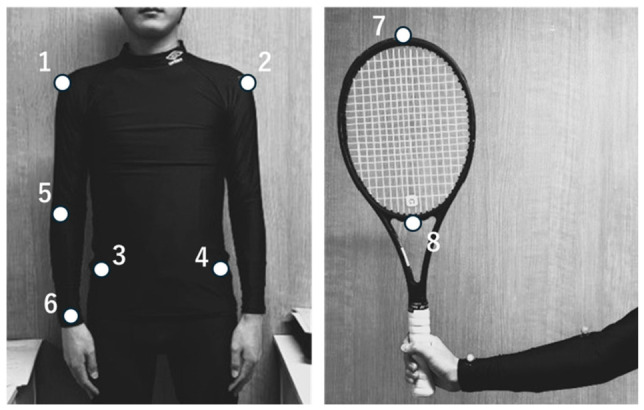
Analyzed segments of the upper-limb and racket. Reflective markers were attached to eight analysis points. The (**left**) and (**right**) figures show the body and racket, respectively. The analysis points comprised eight locations: both acromia (1,2), both iliac crests (3,4), the dominant-side elbow (5), the dominant-side wrist (6), as well as the tip (7) and base (8) of the racket head. These markers were used as the actual points of digitizing. In addition, this study uses automatic tracking for the digitization.

Reflective markers were attached to eight points in total, including six on the participant’s body (both acromiae, both iliac crests, the dominant-side elbow, and the dominant-side wrist) and two on the racket (the tip and base of the racket head) (Figure 2).

For FV, three video cameras (JVC, video camera GC-YJ40) were positioned in front of the participant. Three-dimensional coordinates were obtained from video recordings using a direct linear transformation method. Frame DIAS V (version 2.33, Q’s fix) was used for coordinate digitization. The coordinate system was defined such that the x-axis was parallel to the net and pointed to the participant’s right when facing the opponent’s court; the y-axis pointed toward the net; and the z-axis was perpendicular to both the X- and Y-axes, pointing vertically upward. Calibration was performed using a 2 m rod equipped with a spirit level, and the capture volume was defined as 3 m along the X-axis, 3 m along the Y-axis, and 2 m along the Z-axis. For the BV, three video cameras were positioned in the front-left of the participant, and all other conditions were identical to those for the FV. For left-handed participants, the coordinate systems for FV and BV were reversed relative to those used for the right-handed participants. For analysis, the period from the moment the racket or any body segment began to move to the completion of the backswing was defined as the preparation phase. The period from the moment the racket began to move forward until the ball impacted was defined as the volley phase. The instant of backswing completion was defined as the backswing event, and the instant of ball impact was defined as the impact event.

### 2.4. Measured Variables

The measured variables included the bilateral shoulder rotation angle, pelvic rotation angle, shoulder–pelvis twist angle (defined as the difference between the bilateral shoulder and pelvic rotation angles), and racket forearm angle.

(i) Bilateral shoulder rotation angle

**Figure 3:**
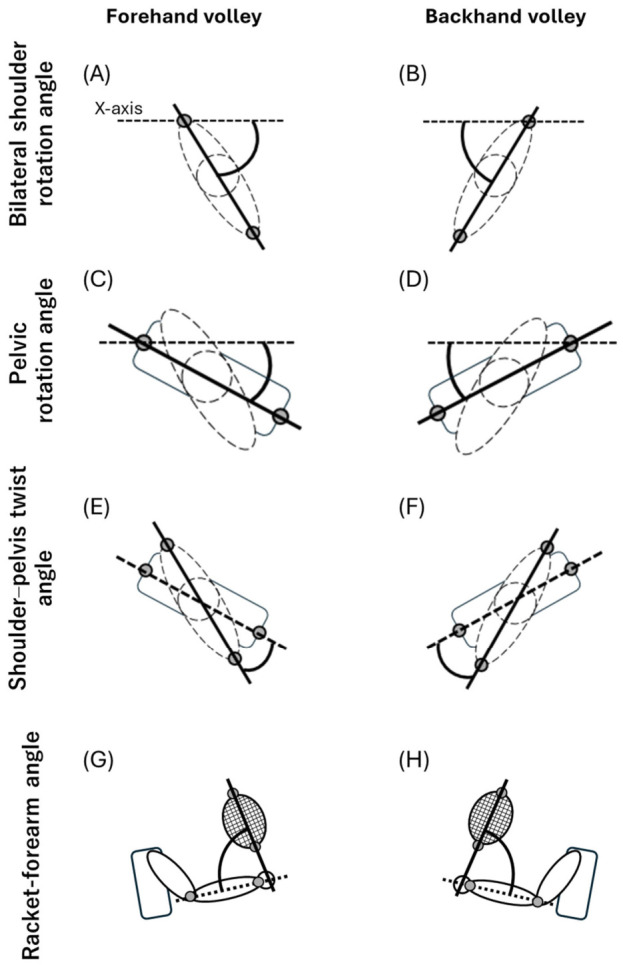
Upper-limb rotation angles. (**A**,**C**,**E**) on the left show the FV, and (**B**,**D**,**F**) on the right show the BV. (**A**,**B**) The bilateral shoulder rotation angle was defined as the angle between the X-axis, which was parallel to the net, and the line segment connecting both acromia. (**C**,**D**) The pelvic rotation angle was defined as the angle between the X-axis and the line segment connecting both iliac crests. (**E**,**F**) The shoulder–pelvis twist angle was defined as the angle between the shoulder line segment and the pelvic line segment. (**G**,**H**) The racket–forearm angle was defined as the angle in three-dimensional space between the vector from the dominant-side wrist to its elbow and the vector from the base of the racket head to its tip.

For the FV, a vector from the left to the right acromion was projected onto the XY plane, and the angle between this vector and the X-axis was defined as the bilateral shoulder rotation angle (Figure 3A).

Using 0° on the x-axis as the reference, rightward rotation of the shoulder vector yielded negative values, whereas leftward rotation yielded positive values. For the BV, the vector from the right to the left acromion was projected onto the XY plane, and the angle between this vector and the X-axis was defined as the bilateral shoulder rotation angle (Figure 3B). This can be considered a 180° rotation of the FV definition. Using 0° on the x-axis as the reference, leftward rotation of the shoulder vector yielded more negative values, whereas rightward rotation yielded positive values. For left-handed participants, the shoulder vector was reversed for both the FV and BV, and the angle between the vector and the X-axis was calculated.

(ii) Pelvic rotation angle

For the FV, a vector from the left to the right iliac crest was projected onto the XY plane, and the angle between this vector and the X-axis was defined as the pelvic rotation angle (Figure 3C). Using 0° on the x-axis as the reference, rightward rotation of the pelvic vector yielded negative values, whereas leftward rotation yielded positive values. For the BV, a vector from the right to the left iliac crest was projected onto the XY plane, and the angle between this vector and the X-axis was defined as the pelvic rotation angle (Figure 3D), which can be considered a 180° rotation of the FV definition. With 0° on the x-axis as the reference, leftward rotation of the pelvic vector yielded more negative values, whereas rightward rotation yielded positive values. For left-handed participants, the pelvic vector was reversed for both the FV and BV, and the angle between the vector and the X-axis was calculated.

(iii) Shoulder–pelvis twist angle

For both the FV and BV, the shoulder and pelvic vectors were projected onto the XY plane, and the shoulder–pelvis twist angle was defined as the value obtained by subtracting the pelvic rotation angle from the bilateral shoulder rotation angle (FV, Figure 3E; BV, Figure 3F). The shoulder–pelvis twist angle defined in this study is an index of the shoulder girdle twisting relative to the pelvis and can be clinically interpreted as one aspect of trunk rotation.

(iv) Racket–forearm angle

When the racket was held in the right hand, the vector from the right wrist to the right elbow was defined as the longitudinal axis of the forearm and the vector from the base of the racket head to its tip was defined as the longitudinal axis of the racket (Figure 3G). When the racket was held in the left hand, the vector from the left wrist to the left elbow was defined as the long axis of the forearm, and the vector from the base of the racket head to its tip was defined as the long axis of the racket (Figure 3H). The racket–forearm angle was calculated in three-dimensional space from the dot product of the two vectors.

### 2.5. Statistical Analysis

Statistical analyses were performed using IBM SPSS Statistics version 26 (IBM Corp., Armonk, NY, USA). For FV and BV, a two-way analysis of variance (ANOVA) was conducted for each measured variable, with player level (skilled vs. less-skilled) as the between-subject factor and time point (backswing event vs. impact event) as the within-subject factor. The significance level was set at 5%.

## 3. Results

**Figure 4:**
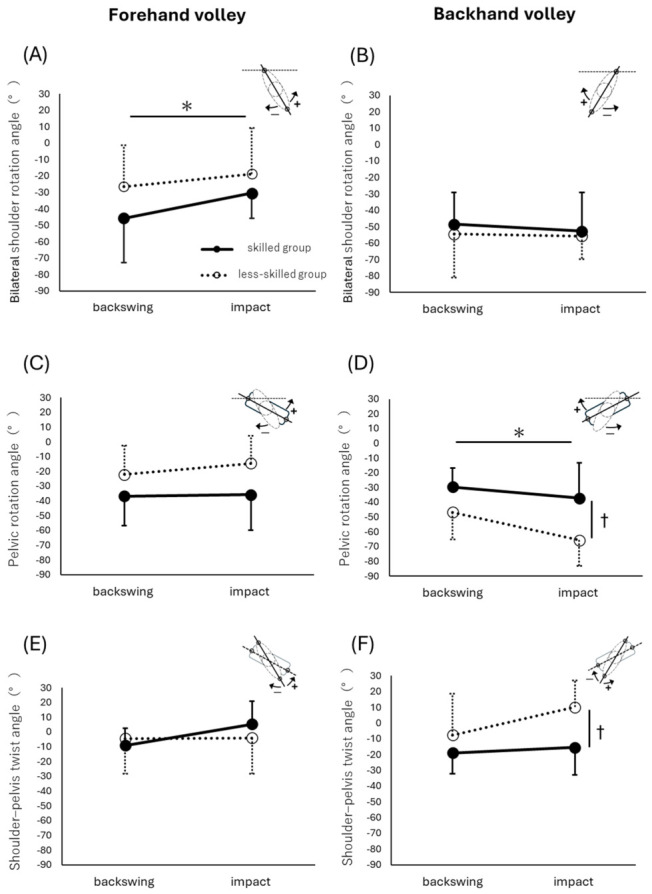
Upper-limb rotation angles at the backswing and impact events. (**A**,**C**,**E**) on the left illustrate the FV, and (**B**,**D**,**F**) on the right show the BV. Solid lines indicate the skilled group, and dashed lines indicate the less-skilled group. An asterisk (*) denotes a significant main effect of player level, and a dagger (†) denotes a significant main effect of time point. For the bilateral shoulder rotation angle, a significant main effect of time point was observed for the FV (**A**), whereas no significant main effects were observed for the BV (**B**). For the pelvic rotation angle, no significant main effects were observed for the FV (**C**), whereas significant main effects of both player level and time point were observed for the BV (**D**). For the shoulder–pelvis twist angle, no significant main effects were observed for the FV (**E**), whereas a significant main effect of player level was observed for the BV (**F**).

**Figure 5:**
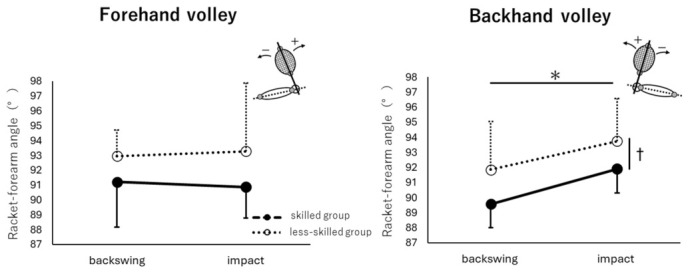
Racket–forearm angles at the backswing and impact events. The (**left**) and (**right**) figures show the FV and BV, respectively. Solid lines indicate the skilled group and dashed lines indicate the less-skilled group. An asterisk (*) denotes a significant main effect of player level, and a dagger (†) denotes a significant main effect of time point. No significant main effects were observed for FV, whereas significant main effects at both the player level and time point were observed for BV.

Figure 4 shows the mean values and standard deviations of the following angles during the backswing and impact events: FV bilateral shoulder rotation angle (Figure 4A), FV pelvic rotation angle (Figure 4C), FV shoulder–pelvis twist angle (Figure 4E), BV bilateral shoulder rotation angle (Figure 4B), BV pelvic rotation angle (Figure 4D), and BV shoulder–pelvis twist angle (Figure 4F). Figure 5 shows the mean values and standard deviations of the racket–forearm angle at backswing and impact events for both the FV and BV.

### 3.1. Forehand Volley

In the FV, as the ball was fed, the shoulders rotated in the reverse direction (rightward rotation) to initiate the backswing (backswing event, skilled group; −45.8 ± 26.9°, less-skilled group; −26.4 ± 25.1°). The shoulders then gradually rotated in the forward direction (leftward rotation) to impact the ball (impact event, skilled group; −30.4 ± 15.3°, less-skilled group; −18.7 ± 27.9°). This movement pattern was the same in both skilled and less-skilled groups. In the FV, the pelvis rotated in the reverse direction (rightward rotation) to initiate the backswing (backswing event, skilled group; −36.7 ± 20.2°, less-skilled group; −22.1 ± 19.5°). The pelvis then underwent little forward rotation (leftward rotation) and impacted the ball (impact event, skilled group; −35.8 ± 24.0°, less-skilled group; −14.6 ± 18.8°). This movement pattern was the same in both skilled and less-skilled groups. The difference between shoulder and pelvic rotation resulted in shoulder–pelvis twisting (backswing event: skilled group; −9.0 ± 11.7°, less-skilled group; −4.4 ± 23.8°, impact event: skilled group; 5.4 ± 15.3°, less-skilled group; −4.1 ± 24.2°).

Accordingly, a two-way ANOVA was performed, with player level and time point as factors. For the bilateral shoulder rotation angle, no significant interaction was found (F_1,18_ = 0.802, *p* = 0.382, η^2^ = 0.043). The main effect of player level was not significant (F_1,18_ = 2.422, *p* = 0.137, η^2^ = 0.119), whereas a significant main effect of time point was observed (F_1,18_ = 7.471, *p* = 0.014, η^2^ = 0.293). For the pelvic rotation angle, no significant interaction was found (F_1,18_ = 0.846, *p* = 0.370, η^2^ = 0.045), and no significant main effects were observed for either player level (F_1,18_ = 4.051, *p* = 0.059, η^2^ = 0.184) or time point (F_1,18_ = 1.397, *p* = 0.253, η^2^ = 0.072). For the shoulder–pelvis twist angle, no significant interaction was found (F_1,18_ = 2.067, *p* = 0.168, η^2^ = 0.103), and no significant main effects were observed for either player level (F_1,18_ = 0.124, *p* = 0.729, η^2^ = 0.007) or time point (F_1,18_ = 2.218, *p* = 0.154, η^2^ = 0.110).

For the racket–forearm angle, both the skilled and less-skilled groups performed the backswing at approximately 90° (backswing event, skilled group; 91.2 ± 3.06°, less-skilled group; 93.0 ± 1.75°) and impacted the ball while maintaining approximately the same angle (impact event, skilled group; 90.9 ± 2.08°, less-skilled group; 93.3 ± 4.60°). No significant interaction was found in the two-way ANOVA with player level and time point as factors (F_1,18_ = 0.142, *p* = 0.711, η^2^ = 0.008), and no significant main effects were observed for either player level (F_1,18_ = 4.290, *p* = 0.053, η^2^ = 0.192) or time point (F_1,18_ = 0.001, *p* = 0.980, η^2^ = 0.000).

### 3.2. Backhand Volley

In the BV, as the ball was fed, the shoulders rotated in the reverse direction (leftward rotation) to initiate the backswing (backswing event, skilled group; −48.5 ± 19.2°, less-skilled group; −54.4 ± 26.4°). The shoulders then underwent little forward rotation (rightward rotation) or reverse rotation (leftward rotation) and impacted the ball (impact event, skilled group; −52.7 ± 23.5°, less-skilled group; −55.6 ± 14.3°). In the BV, the pelvis rotated in the reverse direction (leftward rotation) to initiate the backswing (backswing event, skilled group; −29.6 ± 12.6°, less-skilled group; −46.7 ± 18.4°). The pelvis then continued to rotate in the reverse direction (leftward rotation) to impact the ball (impact event, skilled group; −37.1 ± 24.0°, less-skilled group; −65.7 ± 17.6°). This movement pattern was observed in both groups; however, the less-skilled group showed a greater magnitude in the reverse direction (leftward rotation). This difference between shoulder and pelvic rotation resulted in shoulder–pelvis twisting (backswing event, skilled group; −18.9 ± 13.1°, less-skilled group; −7.7 ± 26.2°, impact event, skilled group; −15.5 ± 17.3°, less-skilled group; 10.1 ± 17.0°).

A two-way ANOVA was performed, with player level and time point as factors. For the bilateral shoulder rotation angle, no significant interaction was found (F_1,18_ = 0.084, *p* = 0.775, η^2^ = 0.005), and no significant main effects were observed for either player level (F_1,18_ = 0.282, *p* = 0.602, η^2^ = 0.015) or time point (F_1,18_ = 0.274, *p* = 0.607, η^2^ = 0.015). For the pelvic rotation angle, no significant interaction was found (F_1,18_ = 2.475, *p* = 0.133, η^2^ = 0.121), but significant main effects were observed for both player level (F_1,18_ = 8.759, *p* = 0.008, η^2^ = 0.327) and time point (F_1,18_ = 13.351, *p* = 0.002, η^2^ = 0.426). For the shoulder–pelvis twist angle, no significant interaction was found (F_1,18_ = 1.254, *p* = 0.278, η^2^ = 0.065). The main effect of time point was not significant (F_1,18_ = 2.715, *p* = 0.117, η^2^ = 0.131), whereas a significant main effect of player level was observed (F_1,18_ = 12.124, *p* = 0.003, η^2^ = 0.402).

For the racket–forearm angle, both the skilled and less-skilled groups performed the backswing at approximately 90° (backswing event: skilled group; 89.6 ± 1.59°, less-skilled group; 91.9 ± 3.21°) and then increased the angle by approximately 2° to impact the ball (impact event: skilled group; 91.9 ± 1.58°, less-skilled group; 93.8 ± 2.83°). No significant interaction was found in the two-way ANOVA with player level and time point as factors (F_1,18_ = 0.104, *p* = 0.751, η^2^ = 0.006), but significant main effects were observed for both player level (F_1,18_ = 6.752, *p* = 0.018, η^2^ = 0.273) and time point (F_1,18_ = 10.213, *p* = 0.005, η^2^ = 0.362).

## 4. Discussion

### 4.1. Bilateral Shoulder Rotation Angle

For FV, the main effect of player level was not significant, whereas a significant main effect of time point was observed, with lower values at the backswing event than at impact. These results suggest that the bilateral shoulder rotation angle followed a similar temporal pattern across player levels. These results suggest that in right-handed players, the FV involves a rightward reverse rotation during the preparation phase, followed by a leftward forward rotation during the volley phase, which is consistent with a previous study [20]. Furthermore, the angles at backswing and impact events in this study were slightly smaller than those previously reported [20]. This difference may be related to the player level of the participants. Whereas the previous study included national tournament-level players in the skilled group and regional tournament-level players in the less-skilled group, the present study included regional tournament-level players in the skilled group and beginners in the less-skilled group.

For BV, no significant main effects were observed at either the player level or time point. This suggests that the bilateral shoulder rotation angle remained relatively unchanged from backswing to the impact event. The bilateral shoulder rotation angle at the backswing event was approximately −50°, suggesting that the BV was prepared by rotating the left shoulder approximately 50° leftward from the initial posture. Subsequently, no further bilateral shoulder rotation occurred, and the racket was moved to impact. This movement pattern may contribute to reducing excessive racket-face variability at impact. This movement pattern may contribute to reducing excessive racket-face variability at impact. In contrast, BV can be regarded as a technique aimed at minimizing unforced errors. Because the impact occurred without shoulder rotation in the forward direction (rightward rotation) after backswing, BV is suggested to involve a more compact motion. These findings may reflect a movement strategy associated with maintaining a compact motion and stable racket during BV. However, because ball speed, accuracy, and success rate were not measured, no direct conclusion can be drawn regarding control or power.

Regarding bilateral shoulder rotation angles, no player-level differences were observed in either the FV or BV. This result suggests that bilateral shoulder rotation may not be a primary factor determining technical proficiency and that this aspect of shoulder movement is less dependent on skill level. However, the less-skilled group in this study consisted of individuals capable of returning the ball over a net under the task conditions. Therefore, caution is required when applying these findings directly to beginners’ initial instructions.

### 4.2. Pelvic Rotation Angle

For FV, no significant main effects were observed at either the player level or time. This suggests that the pelvic rotation angle remained relatively unchanged from the backswing event to impact during FV. In right-handed players, the pelvis rotated in the reverse direction (rightward rotation) at the backswing event, with the right side moving backward and showing few additional angular changes during the volley phase. These findings suggest that during FV, the pelvic girdle functions primarily as a stable foundation for rapid responses to the ball, rather than as a major source of kinetic energy through large rotational movements. Therefore, at least at the player level examined in this study, the pelvic rotation angle itself may not be a key factor determining skill level but rather may represent a common fundamental movement pattern.

For BV, significant main effects were observed at both the player level and time point. In both skilled and less-skilled groups, the values were lower at impact than at the backswing event. In right-handed players, the pelvis rotated in the reverse direction (leftward rotation) at the backswing event and continued to rotate further in the same direction until impact. In the skilled group, the BV appeared to be prepared by approximately 30° of reverse pelvic rotation, which increased to approximately 40° upon impact. In contrast, the less-skilled group showed approximately 45° reverse pelvic rotation at the backswing event, which further increased to approximately 65° at impact. These results indicate that the less-skilled group exhibited greater reverse pelvic rotation than the skilled group at both backswing and impact events. The absence of a player level × time point interaction suggests that the difference in skill level was not specific to one event, but rather reflected an overall difference in the magnitude of pelvic rotation across both events. Therefore, the present findings indicate an overall between-group difference in the magnitude of pelvic rotation across the two analyzed events, with the less-skilled group showing greater reverse pelvic rotation. This should be interpreted as a general kinematic difference rather than an event-specific technical difference.

Overall, these findings suggest that less-skilled players tend to rely on excessive reverse pelvic rotation during BV, whereas higher-level players may place greater emphasis on suppressing pelvic rotation and maintaining postural stability at impact rather than generating power through pelvic rotation.

### 4.3. Shoulder–Pelvis Twist Angle

Shoulder–pelvis twisting is considered important for performance in various sports. In an open-stance forehand stroke in tennis, players increase their trunk rotation during backswing and then unwind it to generate ball speed and power [26]. Similar contributions of trunk or shoulder-girdle rotation to hand, swing, and ball velocities have also been reported in volleyball spiking and baseball batting [27,28].

For FV, no significant main effects or interaction were observed for the shoulder–pelvis twist angle. Although the mean values differed descriptively between groups and between events, these differences were not statistically supported. Therefore, no definitive between-group difference or time-dependent change in shoulder–pelvis twist can be concluded for FV in the present study.

For BV, the main effect of time point was not significant, whereas the main effect of player level was significant. In the skilled group, the shoulders rotated leftward relative to the pelvis during the backswing event, indicating that sufficient twisting had already occurred during preparation. This shoulder–pelvis relationship was largely maintained until impact, suggesting that the ball was struck with a compact motion, likely through the forward translation of the upper limb rather than through additional trunk twisting. In contrast, the less-skilled group showed little shoulder–pelvis separation at the backswing event because the shoulders and pelvis rotated leftward during preparation. However, the pelvis continues to rotate leftward, reaching an impact at impact. This pattern suggests that shoulder–pelvis separation, which had not been established during preparation, may have been produced belatedly. Such delayed twisting may reflect less coordinated trunk movement immediately before impact.

These findings may reflect the temporal constraints of a volley. Volleys are performed over short distances, with limited reaction and swing times. This is believed to stabilize the racket-face orientation and swing path, thereby prioritizing ball control over ball speed [29]. In fact, studies have reported that volleys require rapid responses, with reaction times of approximately 200 ms, and the time from the initial posture to impact varies significantly depending on the ball speed [30]. Taken together, the present results suggest that in both FV and BV, skilled players tend to create greater shoulder–pelvis separation at the backswing event than less-skilled players. In the FV, this twist may be released toward impact, whereas in the BV, it appears to be maintained, with the impact occurring while the shoulder–pelvis relationship is preserved.

### 4.4. Racket–Forearm Angle

For the FV, no significant main effects were observed at either player level or time point, suggesting that both groups maintained a racket–forearm angle of approximately 90° from the backswing event to impact. This common movement pattern may contribute to racket-face stability by minimizing the radial and ulnar deviation of the wrist. Furthermore, no marked differences were observed between the skilled and less-skilled groups. Thus, excessively conscious wrist manipulation during volleying may not be effective. In general, racket-face stability is considered essential in volleys, and this is thought to require a compact motion centered on the forearm and shoulder rather than excessive wrist movement [4,20]. Thus, players may stabilize the racket face by moving the racket and forearm as a single unit rather than by manipulating the wrist independently.

For BV, significant main effects were observed at both the player level and time point. However, the change in angle from the backswing event to impact was only approximately 2° in both groups, and the difference between groups was also approximately 2°, indicating that the magnitude of these differences was small. Thus, although statistically significant differences were detected, the racket–forearm angle was still maintained close to 90° from the backswing event to impact in both groups. Accordingly, even in BV, minimizing radial and ulnar wrist deviations may be important for maintaining racket-face stability.

Taken together, our findings show that players appeared to maintain a racket–forearm angle as close as possible to 90° throughout the volley. It may be important to execute the volley by moving the racket and forearm together while preserving their angular relationship rather than adjusting the racket face primarily with the wrist.

### 4.5. Implications for Coaching

From a practical coaching perspective, the present study does not allow a definitive judgment as to whether individual instructional manuals are entirely correct or incorrect. This is because the study examined kinematic characteristics under limited experimental conditions and did not directly evaluate performance outcomes such as ball speed, shot accuracy, or success rate. Nevertheless, the observed movement patterns in the skilled group suggest that some coaching recommendations are more consistent with the present findings than others. In particular, in the backhand volley, the skilled group showed a more compact movement pattern, with less excessive pelvic rotation, maintenance of the shoulder–pelvis angular relationship until impact, and a racket–forearm angle kept close to 90°. These features may be useful practical cues when coaching the backhand volley. In contrast, coaching instructions that emphasize excessive trunk rotation or a large swing motion in the backhand volley may not necessarily be consistent with the movement characteristics observed in the skilled players in this study.

### 4.6. Limitations and Future Directions

This study has several limitations. First, the definition of skill level and the small sample size should be considered. Both groups in the present study may be regarded as having lower competitive levels, and the number of participants was limited (12 skilled and 8 less-skilled players). These factors may have reduced statistical power and limited the generalizability of the findings.

Second, although ball feeding was performed manually to reproduce conditions closer to actual play, and factors such as ball-feeding speed and contact-point height were controlled as much as possible, some variability may still have occurred. In addition, only one valid trial per participant per condition was analyzed. Because movement variability is inherent in sports performance, particularly in rapid actions such as volleys, this approach may not fully capture within-participant variability and may reduce reproducibility.

Third, the kinematic data were obtained from three video cameras using the direct linear transformation method. Therefore, measurement accuracy may have been influenced by the relatively low sampling rate of the cameras, camera positioning, out-of-plane motion, and digitizing error, particularly when evaluating rapid rotational movements around ball impact.

Fourth, this study examined only selected movement characteristics of middle volleys performed at ball speeds of 50–60 km/h. When responding to faster balls, players may be unable to generate sufficient twist in either the FV or BV, whereas slower balls may allow greater release of the twist before impact, even in the BV.

Future studies should include players at different competitive levels and larger sample sizes, use higher-speed motion capture systems with optimized camera placement, and examine multiple valid trials under more game-like conditions. Such approaches, together with comprehensive analyses combining three-dimensional motion analysis and muscle activity measurements, may allow more precise identification of situation-dependent volley characteristics and determinants of performance.

## 5. Conclusions

This study analyzed the bilateral shoulder rotation angle, pelvic rotation angle, shoulder–pelvis twist angle, and the racket–forearm angle in both skilled and less-skilled players to clarify the movement characteristics during tennis volleys. In the FV, a significant main effect of time point was observed only for the bilateral shoulder rotation angle, suggesting that players used forward rotation (leftward rotation) of the shoulders to generate ball speed and power. In the BV, significant main effects at both the player level and time point were observed for the pelvic rotation angle, and the less-skilled group tended to exhibit excessive reverse rotation (leftward rotation). This suggests that higher skill levels suppress reverse pelvic rotation (leftward rotation) as much as possible and impact the ball from a more stable posture. Additionally, in the BV, a significant main effect of player level was observed for the shoulder–pelvis twist angle, indicating that skilled players tended to establish a twist at the backswing event and impact the ball without releasing this twist, thereby maintaining the shoulder–pelvis angular relationship. This may indicate that skilled players maintain a more consistent shoulder–pelvis angular relationship during BV across the two analyzed events. However, the functional implications of this kinematic pattern for shot control or power remain to be determined. Future studies examining how differences in conditions such as ball speed, contact-point height, and shot direction affect movement are expected to clarify more specific coaching that focuses on both FV and BV.

## Data Availability

The original contributions presented in this study are included in the article. Further inquiries can be directed to the corresponding author.

## Abbreviations

The following abbreviations are used in this manuscript:

FV	Forehand volley
BV	Backhand volley
